# Exploring the Intentions of Jordanian Patients Diagnosed with Hyperlipidemia to Engage in Physical Activity

**DOI:** 10.3390/healthcare13162034

**Published:** 2025-08-18

**Authors:** Ahmad Hussein Al-Duhoun, Maha Atout, Eman Alsaleh, Anees Adel Hjazeen, Majeda M. El-Banna

**Affiliations:** 1 Faculty of Nursing, Mutah University, Al-Karak P.O. Box 61710, Jordan; duhni@mutah.edu.jo; 2 Nursing School, Philadelphia University, Jarash Road, Amman P.O. Box 19392, Jordan; m.atout@philadelphia.edu.jo (M.A.); ealsaleh@philadelphia.edu.jo (E.A.); 3 Royal Medical Services, Amman P.O. Box 855122, Jordan; aneesadel84@yahoo.com; 4 College of Nursing, QU Health Sector, Qatar University, Doha P.O. Box 2713, Qatar

**Keywords:** intention, physical activity, Jordanian patients, hyperlipidemia

## Abstract

Background: The aim of this study was to explore the intention of Jordanian patients diagnosed with hyperlipidemia to engage in physical activity. This objective was achieved via an in-depth analysis of how patient attitudes, subjective norms, and perceived behavioral control can influence patient intentions to exercise. Additionally, this research examined how sociodemographic factors and perceived barriers can impact patient participation in physical activity. Methodology: This study employed a cross-sectional approach on a convenience sample of Jordanian patients diagnosed with hyperlipidemia. To gain the required data, a 15-item questionnaire (derived from the Theory of Planned Behavior) was presented to the participants in the form of an online survey (via several platforms, including WhatsApp, Facebook, and email). Results: The results indicate that perceived behavioral control had a significant correlation with the participants’ intentions to participate in physical activity. Additionally, the findings revealed that there were no significant correlations between demographic features (age, marital status, level of education, and monthly income) and intention to engage in physical activity. However, the results ascertained the existence of several facilitators to exercise (such as financial resource availability, self-interest, beneficial weather conditions, and supportive friends or exercise partners). The most commonly reported barriers to physical activity included time constraints, work commitments, and limitations imposed by existing health conditions. Conclusions: These findings provide valuable insights that can be employed to develop physical activity programs that address the cultural needs of Jordanian patients diagnosed with hyperlipidemia and enhance their levels of physical activity.

## 1. Background

Hyperlipidemia—a condition characterized by excessive levels of lipids (primarily cholesterol and triglycerides) in the bloodstream—is recognized as a significant modifiable risk factor of CVDs [1,2]. It leads to the accumulation of lipids in blood arteries, primarily due to elevated LDL-C levels and diminished HDL-C levels. This results in atherosclerosis [3,4].

According to global health estimates, elevated levels of cholesterol impact approximately 39% of the total adult population and result in millions of deaths annually [3]. Notably, in Jordan, the prevalence of dyslipidemia is significantly higher: the results of a 2019 national cross-sectional survey revealed that 81.6% of adult participants (aged 18–69) had been diagnosed with dyslipidemia; additionally, low high-density lipoprotein cholesterol (HDL-C) was observed in 74.0% of participants [5].

Regular physical activity (PA) is widely acknowledged as a cornerstone treatment in non-pharmacological interventions for controlling hyperlipidemia: it plays a significant role in controlling weight gain, enhancing metabolic function, supporting the long-term regulation of lipid levels, and contributing to overall cardiovascular health [3]. It is highly recommended that patients diagnosed with hyperlipidemia incorporate some form of moderate-intensity physical activity into their daily routine as part of their overall treatment plan [4]. However, despite the established benefits of PA, participation rates remain extremely low. A regional cohort study revealed that only 1.1% of healthy women and 3.5% of men achieved the recommended levels of physical activity [6]. Common obstacles such as insufficient motivation, limited access to facilities, time limitations, and social or cultural norms are attributed to this poor involvement [7,8,9].

Although several Jordanian studies have examined physical activity behaviors in patients with chronic diseases [7,10,11], there is a lack of research concerning the predictors and associated factors of implementing PA among patients with hyperlipidemia.

This study used the Theory of Planned Behavior (TPB) to understand what influences people’s intentions to be physically active, looking at how their attitudes, social pressures, and feelings of control affect their desire to exercise [12,13]. Attitudes refer to an individual’s evaluations of a specific behavior. Subjective norms refer to perceived social pressure. Perceived behavioral control concerns an individual’s perception of the simplicity or complexity of a behavior and their level of control over their performance [14,15].

Therefore, this study aimed to explore the intention to engage in physical activity among Jordanian patients with a diagnosis of hyperlipidemia. Specifically, this research seeks to examine how attitudes, subjective norms, and perceived behavioral control influence patient intentions. Additionally, this study aimed to explore the impact of the relevant sociodemographic factors and perceived barriers to PA.

### Research Questions

1. Are the intentions of Jordanian patients diagnosed with hyperlipidemia to engage in physical activity influenced by specific demographic variables (i.e., gender, age, marital status, level of education, and income)?
2. Do the attitudes, subjective norms, and perceived behavioral controls of Jordanian patients diagnosed with hyperlipidemia predict their intention to engage in physical activity?
3. What underlying beliefs do Jordanian patients diagnosed with hyperlipidemia hold toward physical activity?

## 2. Materials and Methods

### 2.1. Study Design and Sample Population

This study adopted a cross-sectional approach (involving an Arabic-translated questionnaire) to determine whether attitudes, subjective norms, and perceived behavioral control can predict the intentions of Jordanian patients diagnosed with hyperlipidemia to engage in physical activity. The study population was selected through convenience sampling of Jordanian patients with a diagnosis of hyperlipidemia who were eligible for inclusion. The following inclusion criteria were applied to select the sample population: 1. Jordanian patients diagnosed with hyperlipidemia (without other long-term health problems such as diabetes, high blood pressure, or heart disease); 2. participants had to be at least 18 years old; and 3. participants were required to be able to read and write in Arabic. The exclusion criteria included: 1. participants who have any additional long-term health problems such as diabetes, high blood pressure, or heart disease; 2. participants who had a physical or cognitive disability that inhibited their participation in physical activity; or 3. participants who could not read and write in Arabic. The research follows the recommendations of the CHERRIES (Checklist for Reporting Results of Internet E-Surveys) [16]. Supplementary Table S1 contains a completed checklist. The study involved 160 adult participants with hyperlipidemia. The sample comprised both males and females of varying ages, educational backgrounds, and economic levels, effectively representing the target demographic.

### 2.2. Settings

#### 2.2.1. Sample Size Estimation

G*power 3.1 software was employed to compute the sample size via the following sequential approach: F-test was selected from the test family drop-down menu, followed by linear multiple regression, and then fixed model with r-square deviation from zero. Eight predictors—including five demographics and attitudes, subjective norms, and perceived behavior controls—were inserted, and power was set at 0.8 (with a medium effect size of 0.15 and an alpha error of 0.05). Based on accepted norms in social and health sciences research, a power level of 0.80 was selected. This threshold maintains a balance between preserving a manageable sample size and detecting a significant effect, if one exists. This is in line with Cohen [17] suggestions and accepted practice in comparable cross-sectional behavioral research. The minimum required sample size was evaluated as 109; however, this research consisted of 160 cases.

#### 2.2.2. Instrument

The 15-item questionnaire employed by this research was developed by Armitage [18] (as derived from the Theory of Planned Behavior). It employed a 7-point Likert scale to assess the following variables: attitudes towards physical activity, subjective norms of physical activity, perceived behavioral control of physical activity, and physical activity intention [18]. Additionally, the questionnaire included entries concerning the demographic characteristics of the participants (such as age, gender, marital status, monthly income, and level of education). Finally, the questionnaire included four open-ended questions to facilitate an exploration of each participant’s underlying beliefs concerning physical activity. To ensure the linguistic and cultural suitability of the questionnaire, it was translated into Arabic by Kanan, Shahrour, Broome, Bernert, Alibrahim and Hansen [11], who had already translated and adapted it for cultural and linguistic reasons. This version went through cross-cultural validation to make sure it was appropriate for participants from different cultures, such as forward–backward translation and expert validation. It achieved a high content validity index (CVI = 0.97), and formal approval to use the translated questionnaire was obtained from the authors before the commencement of the data collection phase.

#### 2.2.3. Reliability of the Instrument

Cronbach’s alpha (0.87) [18] confirmed the instrument’s reliability and validity. Furthermore, the content validity of the translated questionnaire was assessed as excellent (0.97) [11]. This study employed Cronbach alpha coefficients as definitive criteria to measure the internal consistency of each item. The domain of perceived behavioral control received the highest reliability coefficient (α = 0.920), followed by attitude (α = 0.904) and subjective norms (α = 0.720). The domain measuring intention toward physical activity had the lowest reliability coefficient (α = 0.707). It should be noted that all of the reliability coefficients exceeded the threshold value of 0.70 (see Table 1).

### 2.3. Ethical Considerations

Approval to conduct this study (involving Jordanian patients diagnosed with hyperlipidemia) was obtained from the Institutional Review Board (IRB) at Mutah University (Al-Karak, Jordan).

### 2.4. Participant Recruitment

An invitation letter, outlining the study’s purpose and stressing that participation was anonymous and entirely voluntary, was distributed to each participant. The eligible participants were notified that completing and submitting the electronic questionnaire would be considered as providing their informed consent to participate in the study. Additionally, the cover letter clarified the participants’ ability to withdraw from the study at any time (without penalty or loss of benefits) and verified that any information they provided would be stored securely and confidentially and utilized exclusively for research purposes. Informed consent was obtained via a statement embedded within the questionnaire, which required participants to agree to participate before proceeding. All electronic data were securely stored on the primary researcher’s computer and will be retained for five years (per ethical research guidelines).

### 2.5. Data Collection

The responses of the participants (Jordanian patients diagnosed with hyperlipidemia) were gathered via an electronic self-administered survey (created in Google Forms), which was disseminated via multiple platforms (including WhatsApp, e-mail, and Facebook). Only Jordanian patients with a diagnosis of hyperlipidemia were eligible to participate, despite the survey being distributed electronically. The link to the questionnaire was disseminated via closed patient WhatsApp groups connected to Jordanian healthcare facilities and reliable clinical personnel. To guarantee that all responses came from the target population, participants were also asked to verify their diagnosis and place of residence prior to filling out the survey.

Each participant was sent a cover letter detailing the objectives of the current study, potential risk factors, and the data collection procedures. Additionally, the authors provided the principal investigator’s contact information (email and phone number) to address any inquiries or concerns.

### 2.6. Statistical Analysis

The categorical variables were represented by frequency and percentage, and the scale data were represented by mean, SD, minimum, and maximum. Spearman’s rho correlation was employed to explore the relationship between participant age and intention to engage in physical activity. An independent test and one-way ANOVA were utilized to investigate mean differences in dependent variables according to demographic characteristics. Additionally, a multiple linear regression was conducted to predict intention toward physical activity as a function of attitude, subjective norms, and perceived behavioral control. A *p*-value of less than 0.05 was deemed to be statistically significant, and IBM SPSS Statistics (Version 27) was employed to analyze the data.

### 2.7. Qualitative Data Analysis

We used conventional content analysis, according to Hsieh and Shannon [19], to look at the qualitative part of this study. Two members of the research team looked over the answers given by the participants. Independently, codes were created and looked at in an inductive way. After that, the resulting codes were grouped into categories that showed what they all meant. Then, the number of answers in each category was counted and shown as descriptive statistics. When there was a disagreement about how to code and categorize the themes, it was settled by discussing them to ensure the results were honest and clear.

## 3. Results

A total of 160 participants with hyperlipidemia participated in the study. The ages of the participants ranged from 23 to 87 years old, with a mean of 49.98 (SD = 12.43). The gender characteristics of the participants are as follows: 58.8% (n = 94) of the study population were male, and 41.3% (n = 66) were female. An examination of the marital status of the participants revealed that 91.3% (n = 146) of them were married, and 8.8% (n = 14) were unmarried. Regarding educational attainment, 62 participants (38.8%) held a bachelor’s degree, while 25.0% (n = 40) had received only a secondary education (or less). Concerning income, nearly one-third of participants (31.3%, n = 50) reported earning between 501 and 1000 Jordanian Dinars (JD) per month; 26.3% (n = 42) earned between 301 and 500 JD per month; and 18.8% (n = 30) earned less than 300 JD per month. These sample characteristics are summarized in Table 2.

### 3.1. Description of the Physical Activity Domains Included in the Questionnaire

The questionnaire concerning physical activity consisted of four domains: three of these domains (attitude, subjective norms, and perceived behavioral controls) served as independent variables, while the fourth domain (intention towards physical activity) was employed as a dependent variable. The descriptive statistics detailed in Table 3 (below) reveal that the mean for attitude was 35.26 (SD = 6.60) and that the score for this domain ranged between 6 and 42. The results for subjective norms ranged from 7 to 21 (M = 15.98, SD = 3.26), and the results for perceived behavioral control towards physical activity ranged from 4 to 28 (M = 18.91, SD = 6.19). The dependent variable (intention toward physical activity) recorded a mean score of 11.01 (SD = 2.52) with scores that ranged between 4 and 14.

#### Answering the Research Questions

(Q1) Are the intentions of Jordanian patients diagnosed with hyperlipidemia to engage in physical activity influenced by specific demographic variables (i.e., gender, age, marital status, level of education, and income)?

To address this research question, the results presented in Table 4 (below) reveal that none of participant age, marital status, education level, or monthly income exhibited a statistically significant association with their intention to engage in physical activity (*p* > 0.05 for all variables).

- Age: r_s_ = −0.050, *p* = 0.533
- Gender: t = 0.052, *p* = 0.958
- Marital status: t = 0.684, *p* = 0.495
- Education level: F = 0.132, *p* = 0.941
- Monthly income: F = 0.103, *p* = 0.958

(Q2) Do the attitudes, subjective norms, and perceived behavioral controls of Jordanian patients diagnosed with hyperlipidemia predict their intention to engage in physical activity?

To address this research question, this study employed multiple linear regression.

The results presented in Table 5 indicate that the overall regression model with three predictors was statistically significant—*F* (3.156) = 38.875, *p* < 0.001—and accounted for 41.7% of the total variability in intention toward physical activity score. In terms of individual effect, perceived behavioral control had a statistically significant impact on intention toward physical activity (*B* = 0.229, *t* = 7.823, *p* < 0.001), which indicates that for every additional unit of perceived behavioral control, the participants’ intentions are expected to increase by an average of 0.229 units (holding the other variables constant). Additionally, the attitude toward physical activity predictor demonstrated a marginally significant impact (B = 0.057, t = 1.934, *p* = 0.055); however, the subjective norms of physical activity did not demonstrate any significant results (B = 0.007, t = 0.142, *p* = 0.887).

(Q3) What underlying beliefs do Jordanian patients diagnosed with hyperlipidemia hold toward physical activity?

To address research question 3, this research employed the following open-ended questions (each of which is designed to facilitate an exploration of a specific area):

1. (To investigate participant advantages of physical activity): What are your perceptions of the benefits of participating in regular physical activity?
2. (To evaluate societal pressure): Are you aware of any individuals or groups (significant to you) who would disapprove of your participation in regular physical activity? If so, how do they express their disapproval?
3. (To evaluate enabling factors): What facilitates your engagement in regular physical activity?
4. (To identify barriers): What factors preclude your engagement in regular physical activity?

Participants’ responses concerning the perceived benefits of physical activity, social pressure, facilitating factors, and barriers to engaging in physical activity were analyzed using content analysis. 147 (91.9%) participants responded to the first open-ended question concerning the perceived benefits of physical activity. 86.4% of these responses indicated a belief that physical activity provides significant health benefits (including lowering blood pressure, reducing lipid levels, decreasing blood sugar, and minimizing the risk of cardiovascular diseases). The second open-ended question (societal pressure) received 142 (88.8%) answers. 95% of the respondents reported that important individuals in their lives (such as family members, friends, or healthcare providers) supported their engagement in physical activity. 124 (77.5%) of participants responded to the third open-ended question concerning the factors that may facilitate their engagement in physical activity. These responses identified several factors, including financial resource availability (i.e., the ability to afford gym memberships or exercise equipment), the support of friends or exercise partners, personal interest, and advantageous weather conditions. The majority of respondents (71%) identified the following elements as key facilitators for physical activity: lack of time constraints, easy access to exercise facilities, and good health. Additional factors mentioned included financial ability, social support, personal interest, and favorable weather conditions (29%). The fourth open-ended question (perceived barriers to engaging in physical activity) received 150 (93.8%) responses. The most frequently reported barriers were lack of time, health constraints, and work commitments (79.3%). Other participants mentioned the lack of suitable exercise facilities and family responsibilities as limiting factors (16.7%). A small percentage of respondents (4%) reported experiencing no barriers to physical activity (see Table 6).

## 4. Discussion

The primary objective of this research concerned an examination of the intent of Jordanian patients diagnosed with hyperlipidemia to engage in physical activity. Specifically, this study was designed to analyze how attitudes, subjective norms, and perceived behavioral control impact patients’ intentions to engage in physical activity. Additionally, this research scrutinized the influence of sociodemographic factors and the perceived barriers to engagement in physical activity.

The findings indicate that there is no significant correlation between demographic factors (such as age, marital status, level of education, and monthly income) and the participants’ intentions to participate in physical activity. However, the existing literature has consistently reported that an individual’s level of physical activity gradually declines as they age [20,21]. Additionally, recent research has revealed that a higher level of education correlates with elevated levels of physical activity [22]. Individuals with higher levels of education demonstrated an increased awareness of the benefits of physical activity; therefore, education should be recognized as a facilitator of physical activity that can be incorporated into personalized physical activity interventions to increase participation. Typically, individuals with higher levels of education are more physically active and display an enhanced understanding of their overall health. One possible explanation for the finding of non-significant correlations between demographic characteristics and the intention to engage in physical activity is that the diagnostic similarities among the patients may have resulted in common health concerns and lifestyle objectives, rather than being affected by other sociodemographic characteristics such as age, marital status, educational attainment, and monthly income. Moreover, as these individuals live in Jordan, they may be influenced by information from similar sources, such as social media and other informational outlets, that significantly impact their intention to engage in physical activity. Moreover, traditional views concerning health perceptions and the management of chronic illnesses may exert a more significant influence on the participants than sociodemographic factors alone. The selection bias inherent in this study’s convenience sampling method may result in a discrepancy between current research and its outcomes. Future research may require the implementation of stratified or random sampling methods to achieve a greater grasp of the variability of these factors.

The findings of this research indicate that perceived behavioral controls concerning physical activity are significant predictors for the level of physical activity among Jordanian patients with hyperlipidemia: an individual’s perception of their ability to participate has a distinct influence on their intention to engage in physical activity. The Theory of Planned Behavior (which serves as the foundation for this research) posits that these variables can predict an individual’s health behaviors by identifying their motivations to perform physical activity [23]. This aligns with previous research that has demonstrated the influence of such variables in predicting the health behaviors (such as physical activity [12,13,24] and healthy eating [25] of both the healthy and the chronically ill. Additionally, a study involving patients with coronary artery diseases identified a significant correlation between behavioral control and the participants’ intentions to engage in physical activity [11]. However, it should be noted that subjective norms (derived from social environmental perceptions that influence an individual’s decision to engage/disengage with the behavior) were not significant predictors of physical activity among the participants.

This study makes a unique contribution to the existing literature for multiple reasons. Numerous global research studies have employed the theory of planned behavior to investigate physical activity among patients with different chronic diseases [12,13,24]. However, little study has utilized the theory of planned behavior to examine the intentions for physical exercise among high-risk patients, specifically those with hyperlipidemia in the Middle East. For instance, Alsaleh and Baniyasin [7] and Kanan, Shahrour, Broome, Bernert, Alibrahim and Hansen [11] investigated physical activity among Jordanian patients with cardiovascular disease, whereas there is no reported research addressing the intention to engage in physical exercise among patients with hyperlipidemia in Jordan. Second, this study identified perceived behavioral control as the primary predictor of the desire to engage in physical exercise, surpassing the impact of attitudes and subjective norms related to physical activity. This finding is consistent with numerous international studies indicating that self-efficacy-related characteristics are the most powerful motivators for adopting health behaviors [12,13]. Finally, this study incorporated interviews to investigate the cultural obstacles and support associated with physical activity, providing a comprehensive understanding of how patients with hyperlipidemia engage in exercise.

Via their responses to the open-ended questions, the participants in this research reported that social support (provided by friends, family, or healthcare professionals) played a pivotal role in facilitating their participation in physical activity. The findings of the current study revealed that the majority of participants understood the advantages of physical activity and its role in lowering blood pressure, reducing lipid levels, decreasing blood sugar levels, and minimizing the risk of cardiovascular disease. Additionally, they recognized that several factors (such as time availability, personal interest, access to physical activity, and social support from friends, family, and healthcare professionals) are key drivers for encouraging them to engage in physical activity, and this aligns with previous studies involving healthy adults [26,27]. These findings emphasize the importance of incorporating such facilitators into individualized PA interventions to encourage individuals to participate in regular physical activity.

In this study, the most frequently reported barriers to physical activity were time constraints, health restrictions, work commitments, family responsibilities, and a lack of exercise facilities, all of which align with previous research involving healthy adults [26,27] and chronically ill patients. These barriers to physical activity could be addressed via their inclusion in PA programs. For example, one prospective cohort study (consisting of 846 patients who had undergone coronary artery bypass grafting) reported that participants who had access to local green spaces (such as parks, public gardens, and playing fields) exhibited a 52% increased probability of engaging in physical activity [28]; therefore, increasing access to green spaces may play a pivotal role in encouraging patients with hyperlipidemia to engage in sustained physical activity.

By establishing additional community sports facilities and venues, the government can create a conducive environment that fosters and develops the physical activity habits of patients with chronic illnesses (such as hyperlipidemia).

The theory of planned behavior has been applied in several research studies on patients with different chronic health conditions. These studies have shaped a number of global health policy strategies. For example, many governments throughout the world have established cardiac rehabilitation programs such as educational sessions, behavioral coaching, and peer support to lessen the chance of having heart disease as part of their goals to stop diseases from spreading [29,30]. There is no proof in Jordan that TPB policies work for people with hyperlipidemia; however, a few heart disease research studies in Jordan, such as Alsaleh and Baniyasin [7] and Kanan, Shahrour, Broome, Bernert, Alibrahim and Hansen [11], have proposed recommendations for future culturally sensitive interventions such as self-efficacy, social support, and community-based interventions. Therefore, in Jordan, the findings of the current study might be used to improve public health policies that encourage people with hyperlipidemia to be more active.

### Practical Implications

This research provides a significant opportunity to reconsider and amend the existing approach to encouraging physical activity among patients diagnosed with hyperlipidemia. Given that several participants perceived their capacity for physical activity to be influenced by their sense of control, it is logical to concentrate on techniques that enhance confidence and mitigate daily obstacles. Practical measures (such as developing home-based exercise regimens, providing adaptable guidance for physical activity during routine medical consultations, or assisting patients in establishing reasonable and attainable objectives) could significantly impact health outcomes. The findings indicate the need to introduce initiatives and settings that facilitate and enhance the appeal of physical activity, as well as improve the autonomy and self-confidence of individuals in managing their own physical activities within the Jordanian community. This may involve improving access to safe and affordable public spaces and offering culturally relevant exercise programs, including those that promote gender sensitivity, such as women-only gym sessions. Additionally, culturally appropriate educational materials should be developed to encourage patients, families, and friends to engage in regular physical activities. Furthermore, regular activity could be enhanced by sending encouraging messages through smartphones that include information about sports support programs. These interventions aim to lower practical and mental barriers, give people more power, and be in line with the religious and social values that affect health behaviors in Jordan.

The current study demonstrates significant predictive value for perceived behavioral control, indicating the need to bolster patients’ confidence in their ability to participate in physical activity. This may be achieved by alleviating any medical or psychological problems that diminish their confidence. Employing a smartphone application to distribute concise, inspirational videos or messages to promote physical activity could be a useful approach. Moreover, culturally tailored strategies could enhance patient involvement in home exercise, especially for women who are unable to participate in outdoor physical activities. Promoting family-oriented activities is crucial to augment patients’ motivation for engaging in physical exercise. Healthcare practitioners aim to motivate their patients to set realistic activity goals to overcome their fears and anxieties.

## 5. Limitations

The current study contains several limitations. The first limitation concerns the study’s adoption of a convenience sampling method for participant selection, which restricts the ability to generalize its findings to a wider population; therefore, this cross-sectional study must be approached with caution. Furthermore, since this study is not experimental, it is not possible to definitively conclude whether attitude or perceived behavioral control influences patients’ intentions to engage in physical activity. Therefore, future studies should utilize probability-based or stratified sampling approaches in various healthcare settings. This will enhance the external validity of the findings and reinforce the evidence used to guide public health strategies.

The second limitation arises from the fact that all of the measurements were derived from self-reporting (rather than direct observation), and this may have resulted in a degree of inaccurate recall and reporting and social reporting biases [31]. Therefore, in order to increase the accuracy of measurement, future studies should use objective measures of physical activity, like accelerometers or fitness trackers, which could also give us more accurate and reliable data on how active the participants are. We also recommend longitudinal designs to observe how TPB structures alter behavior over time.

Finally, although this cross-sectional study reveals connections, it does not establish cause-and-effect relationships, which constrains its capacity to ascertain whether any specific element has a direct influence on modifying physical activity behaviors.

## 6. Conclusions

This study revealed that perceived behavioral control was the strongest predictor of physical activity intentions among hyperlipidemic patients, whereas attitudes had a minimal effect, and subjective norms had no effect at all. The qualitative findings indicated that sufficient financial resources, supportive friendships, favorable weather conditions, sufficient time, and availability of appropriate exercise locations were all significant factors facilitating physical activity. Conversely, the primary challenges included time constraints, health concerns, professional responsibilities, insufficient exercise facilities, and familial obligations. The findings of this study indicate that culturally appropriate support programs should be initiated to enhance individuals’ activity levels, reinforce their confidence, and facilitate their participation.

## Figures and Tables

**Table 1:** Instrument reliability of the study (n = 160).

Variables	Number of Items	Cronbach’s Alpha
Attitude	6	0.904
Subjective norms	3	0.720
Perceived behavioral control	4	0.920
Intention toward physical activity	2	0.707

Abbreviations: Cronbach’s alpha = measure of internal consistency.

**Table 2:** Participant Demographics (mean ± SD).

Variables	Category	Frequency	Percentage	Mean (SD)
Age in years				49.98 (12.43)
				Range 23–87
Gender	Male	94	58.8	
	Female	66	41.3	
Marital status	Married	146	91.3	
	Unmarried	14	8.8	
Education level	Secondary or less	40	25.0	
	Diploma	30	18.8	
	Bachelor	62	38.8	
	Postgraduate	28	17.5	
Monthly income level	Less than 300 JD	30	18.8	
	Between 301 and 500 JD	42	26.3	
	Between 501 and 1000	50	31.3	
	More than 1000 JD	38	23.8	

Note: SD = Standard Deviation.

**Table 3:** Descriptive statistics for domains concerning physical activity.

Variables	Min	Max	Mean	SD
Attitude	6.00	42.00	35.26	6.60
Subjective Norms	7.00	21.00	15.98	3.26
Perceived behavioral control	4.00	28.00	18.91	6.19
Intention toward physical activity	4.00	14.00	11.01	2.52

Abbreviations: SD = standard deviation.

**Table 4:** Association between participant demographic variables and intention to engage in physical activity (N = 160).

Variables	Category	Mean	SD	Test Value	*p*-Value
Age in years				−0.05	0.533 ^rs^
Gender	Male	11.02	2.64	0.052	0.958 ^t^
	Female	11.00	2.36		
Marital status	Married	11.05	2.50	0.684	0.495 ^t^
	Unmarried	10.57	2.82		
Education level	Secondary or less	11.03	2.53	0.132	0.941 ^F^
	Diploma	11.20	2.52		
	Bachelor	10.87	2.72		
	Postgraduate	11.11	2.13		
Monthly income level	Less than 300 JD	11.20	2.01	0.103	0.958 ^F^
	Between 301 and 500 JD	10.93	2.82		
	Between 501 and 1000	11.06	2.72		
	More than 1000 JD	10.89	2.33		

Abbreviations: rs = Spearman’s rho correlation; t = independent *t*-test; F = one-way ANOVA.

**Table 5:** Predicting intention toward physical activity as a function of attitude, subjective norms, and perceived behavioral control (N = 160).

Predictors	Unstandardized Coefficients		Standardized Coefficients	T	Sig.	95% CI
	B	Std. Error	Beta			
Attitude toward physical activity	0.057	0.030	0.150	1.934	0.055	−0.001, 0.116
Subjective norms of physical activity	0.007	0.052	0.009	0.142	0.887	−0.110, 0.095
Perceived behavioral control of physical activity	0.229	0.029	0.563	7.823	<0.001	0.171, 0.287

Model summary: F(3,156) = 38.875, *p* < 0.001, R^2^ = 0.428, Adjusted R^2^ = 0.417. Abbreviations: B = unstandardized coefficient; SE = standard error; CI = confidence interval; t = *t*-test value.

**Table 6:** Perceived beliefs toward physical activity (n = 160).

Question	Factors	n	%
Perceived benefits (n = 147)	Health benefits	127	86.4
	General Benefits	20	13.6
Social pressure (n = 142)	It is important that individuals approve of physical activity.	135	95
	It is important that individuals disapprove of physical activity	7	5
Facilitating factors (n = 124)	Suitable time, place, and good health	88	71
	Availability of money, friends and interest, and suitable weather	36	29
Barriers (n = 150)	Lack of time, health constraints, and work commitments	119	79.3
	Lack of suitable facilities, family responsibilities	25	16.7
	No barriers	6	4

Note: Percentages reflect response proportions based on each open-ended question.

## Data Availability

The data presented in this study are available on request from the corresponding author.

## Supplementary Materials

The following supporting information can be downloaded at: https://www.mdpi.com/article/10.3390/healthcare13162034/s1, Supplementary Table S1: Checklist for Reporting Results of Internet E-Surveys. Reference [16] has been cited in the Supplementary Materials.
